# Energy Requirements for Older Patients with Type 2 Diabetes: A Narrative Review of the Current Findings and Future Tasks

**DOI:** 10.3390/nu13030753

**Published:** 2021-02-26

**Authors:** Fuminori Katsukawa

**Affiliations:** Sports Medicine Research Center, Keio University, Yokohama 223-8521, Japan; fuminori@keio.jp; Tel.: +81-45-566-1090

**Keywords:** diabetes mellitus, older patients, energy expenditure, body mass index, doubly labeled water, low-intensity physical activity

## Abstract

Aging patients with type 2 diabetes (T2DM) have a high risk of frailty and disability. This narrative review summarizes the current findings and future tasks regarding the following issues: (1) the optimum body mass index (BMI) target for patients with T2DM, (2) energy expenditure and requirements of patients with T2DM, and (3) the significance of low-intensity physical activity (LPA) as a key determinant of total energy expenditure (TEE) among the older population. While weight reduction is associated with an improvement in glycemic control, an increased risk of mortality as well as disability related to unhealthy BMI must also be considered, especially in older patients. The optimum BMI range and energy requirements for maintaining a healthy BMI should be identified. Limited evidence has shown that the TEE measured using the doubly labeled water method in patients with T2DM did not differ from that of subjects without diabetes, suggesting that the energy requirement data of subjects without diabetes may be applicable to these populations. LPA is one of the determinants of variability in the energy requirements of older patients with T2DM, and the beneficial effects of increasing LPA on nutritional intake and frailty prevention should be investigated further.

## 1. Introduction

Type 2 diabetes mellitus (T2DM) is associated with an increased risk of loss of muscle mass and strength, which will eventually lead to frailty and disability in older patients [1]. Recently, it has been demonstrated that hyperglycemia promotes muscle atrophy in diabetic animal models [2], and multimodal management, including adequate energy intake, physical activity, and improved glycemic control, is key to preventing the progression to disability [3]. The purpose of this review is to summarize the current findings and future tasks regarding the following issues: (1) the optimum body mass index (BMI) target for patients with T2DM, (2) energy expenditure and requirements of patients with T2DM, and (3) the significance of low-intensity physical activity (LPA) as a key determinant of total energy expenditure (TEE) among the older population.

## 2. Optimum BMI Target in T2DM

A systematic review of weight loss interventions revealed a linear relationship between weight loss and HbA1c reduction, with an HbA1c reduction of 0.1% for each 1 kg of weight lost in the entire population [4]. Another clinical weight reduction trial (DiRECT trial) showed that the rate of “remission”, defined as an HbA1c level of <6.5% after ≥2 months off all antidiabetic medications, increases with greater weight loss [5]. Moreover, a post hoc analysis of the Look AHEAD trial showed that the ≥10% weight loss observed in the first year of the study was associated with a significant reduction of cardiovascular disease outcomes [6]. These data suggest that weight reduction is associated with the improvement of glycemic control, withdrawal of antidiabetic medications, and reduction of cardiovascular complications in patients with T2DM.

In contrast, epidemiological observational studies have demonstrated a J-shaped association between BMI and mortality in general [7] and diabetic populations [8,9]. A pooled meta-analysis of 239 prospective studies in the general population showed this J-shaped relationship, and the BMI associated with the lowest mortality shifted to a higher range with increasing age [7]. A meta-analysis of 24 cohorts with T2DM also revealed this J-shaped relationship, with the lowest all-cause mortality risk associated with a BMI of 31–35 and 28–31 for men and women, respectively [8]. In this study, the increased mortality risk associated with a high BMI was not statistically significant in men. However, the risk associated with a low BMI was prominent in both sexes, and this finding has been confirmed by another cohort study of patients with diabetes [9]. This reduced risk of mortality (“obesity paradox”) has also been reported in various comorbidities often associated with T2DM in older patients, such as coronary heart disease [10], hypertension [11], and heart failure [12]. In contrast, a recent meta-analysis examining the relationship between BMI and the risk of disability among older general populations [13] has reported that individuals with a BMI of >28.0–32.9 and ≥33.0 were 19% and 43% more prone to disability risks compared to those in the reference group (BMI: 23.0–23.9), respectively. A BMI range of 24–28 presented a decreasing risk of disability compared with the reference BMI. Although these findings remain to be confirmed in patients with T2DM, the increased disability risk associated with a higher BMI must also be considered in the older population.

In the systematic reviews of obesity guidelines from the American College of Cardiology/American Heart Association/The Obesity Society [14], the weight reduction pattern with energy-restricted dietary intervention has been summarized as follows: (1) maximum weight loss of 4–12 kg was achieved after 6 months, and thereafter (2) slow weight regain was observed, with a total weight loss of 4–10 kg at 1 year and 3–4 kg at 2 years. These reviews have acknowledged the importance of sustained weight loss of 3–5% in producing clinically meaningful health benefits, although greater weight losses (5–10%) produce greater benefits.

Thus, while weight reduction is effective for metabolic control, only a modest weight loss of 3–5% is sustainable with dietary intervention, and the mortality risk associated with a lower BMI as well as the disability risk associated with a higher BMI must also be considered. Optimum BMI ranges should be considered not only in terms of mortality but also disability in older patients, and more data are needed to determine the optimal BMI ranges for older patients with T2DM.

## 3. Alterations in Energy Expenditure in T2DM

Currently, estimates of energy requirements are based on measurements of energy expenditure. This is partly due to dietary assessment methods underestimating habitual energy intake, which is also true for patients with T2DM [15,16,17,18]. In these studies, the reported energy intake (rEI) was determined using 3-day dietary recall [15], 3-day dietary record [16,17], or 3-day mobile phone image-based dietary record [18] along with simultaneous measurement of TEE using the doubly labeled water (DLW) method. The results of these studies have shown that the rEI underestimates habitual energy intake by 10–56% on an average, and the relatively large standard deviation values of rEI/TEE ratio (13–20%) suggest individual differences in the severity of underreporting.

In order to evaluate the energy requirements for patients with T2DM, alterations in each component of TEE are discussed below. The basal metabolic rate (BMR) of patients with T2DM has been measured either using a ventilated hood system [15,19,20,21], by putting on a face mask [22,23], or during a stay in the respiratory chamber [24,25,26]. The results indicated that the BMR of patients with T2DM either showed no difference [15,19,22] or was 5–7% higher [20,21,23,24,25,26] than that of patients without T2DM. This increased energy expenditure may be due to increased hepatic glucose production [27]. In fact, some studies have indicated that a higher fasting plasma glucose concentration is associated with a higher BMR [23,28,29]. Other studies have shown that both exogenous [27,29,30] and endogenous insulin (C-peptide immunoreactivity after intravenous glucagon injection) [30] have an impact on lowering the BMR of patients with T2DM, probably mediated by the suppression of glucose production by insulin. Another possible mechanism involved in raising the BMR is increased renal glucose reabsorption, which is an energy-dependent process [31]. Hyperglycemia increases the amount of glucose filtered by the glomerulus and, consequently, tubular glucose reabsorption is increased [32]. In addition, comorbidities often associated with T2DM in older patients, such as subclinical inflammation [33], hepatic steatosis [34], hypertension [35], and heart failure [36], may also contribute to the raised BMR.

In contrast to BMR, the thermic effect of food (TEF) has been reported to be decreased in patients with T2DM [37,38]. Golay et al. [37] measured the energy expenditure during the 3 hours after a 100 g oral glucose load and found that the two groups of obese patients with T2DM (increased and reduced insulin responses) showed a lower TEF than the controls without diabetes. Segal et al. [38] also compared TEF during the 3 hours after a 720 kcal test meal between lean and obese men without diabetes and men with T2DM. They found that the TEF was greater for the lean men than for the obese men and greater for the obese men than for the men with T2DM. After an acute bout of exercise, TEF for the obese men and the men with T2DM increased, and after 12 weeks of exercise training, TEF for the men with T2DM increased further after acute exercise; however, these values were not normalized and were still lower than those of the lean men. The authors suggested that impaired activation of the sympathetic nervous system [39] and reduced insulin sensitivity [37] may have contributed to the decrease in TEF.

Activity-related energy expenditure is a variable component of TEE. Physical inactivity is a well-established risk factor for developing T2DM [40]. In contrast, both cross-sectional and longitudinal studies have shown that T2DM increases the risk of physical disabilities, including difficulties with activities of daily living (ADL) and instrumental ADL and mobility limitations among older patients [41], which will consequently reduce physical activities. Studies that evaluated physical activity in patients with and without T2DM have unanimously reported that patients with T2DM had lower levels of physical activity than controls without diabetes, regardless of whether subjective (questionnaire) or objective (accelerometer) measures of physical activity were used [42]. During their stay in the respiratory chamber, patients with T2DM were reported to have a higher 24-hour energy expenditure than controls without diabetes [24,26]. These results may be affected by the confined settings of the chamber where physical activities of both controls and patients were modified.

There are a limited number of studies in which free-living TEE was measured using the DLW method in patients with T2DM [15,16,43,44,45,46] (Table 1). These studies included patients treated with either insulin [43,45], oral antidiabetic agents [15,16,43,44,45,46], or diet [16,43,45,46]. The study by Chong et al. [44] reported that the addition of metformin in six patients already on insulin did not lead to any measurable changes in TEE. Three studies that included controls without diabetes showed that there was no difference in TEE between patients with and without T2DM [15,16,45]. Another study that compared patients with T2DM to those with impaired glucose tolerance/impaired fasting glucose and those with normal glucose tolerance found no difference between the three groups [46]. Based on this limited evidence, the energy requirement values of subjects without diabetes may be applicable to those with T2DM. However, further data are required in this regard.

## 4. Individual Variability of Energy Requirements and Its Possible Determinant Factors

According to the studies that used the DLW method, patients with T2DM had comparable TEE values to those without diabetes. However, this does not necessarily mean that the energy requirements of all patients with T2DM are equal. In fact, one of these studies [45,47] found that the individual variability in the patients’ physical activity level (PAL = TEE÷BMR) was almost as large as that in the general population [48]. In Table 2, the patients with T2DM were classified into three groups according to their PAL values. The data indicate that the TEE of the patient with the highest PAL value was approximately 500 kcal/day higher than that of the patient with the lowest PAL value, and the accelerometry data showed that the difference was mainly attributed to the time spent doing non-locomotive daily activities, especially LPA.

These findings were replicated in the studies of Bastone et al. [49,50], where community-dwelling frail and non-frail subjects (66–86 years of age) were evaluated. Their accelerometry data showed that both the frail and non-frail subjects spent > 96% of their time doing either sedentary activity or LPA. Compared to the frail subjects, the non-frail group spent approximately 140 additional minutes doing LPA instead of sedentary activity [49], which eventually corresponds to a difference of approximately 600 kcal/day of TEE measured using DLW [50]. The results from these studies suggest that daily LPA is a key determinant of TEE and, therefore, energy requirements in older subjects.

Despite the difference in TEE (Table 2), the HbA1c showed no difference between the three T2DM groups, suggesting that the energy expenditure related to doing LPA was not associated with an improvement in glycemic control. Similar findings were reported in another study of patients with T2DM, where isotemporal substitution of each 30 minutes per day of LPA with sedentary activities was not associated with any improvement in fasting plasma glucose values [51]. However, an increase in TEE is associated with an increased intake of energy as well as other nutrients. In older subjects, reduced muscle protein synthesis in response to protein intake has been observed [52], and this “anabolic resistance” will theoretically increase protein requirements. The protein intake, estimated from the TEE and protein–energy percentage obtained using a 3-day food diary, was sufficient to meet the recommended dietary allowance in each group (Table 2), but subjects with a higher PAL value may be at a higher advantage regarding maintenance of muscle mass and frailty prevention.

Patients with T2DM spend most of their time either being sedentary or doing LPA, and the time spent doing moderate-to-vigorous physical activity is minimal [51,53]. LPA is a mixture of various involuntary daily activities. The effect of increasing LPA on the improvement in the nutritional intake of older subjects along with the classification and evaluation of this activity should be investigated further.

## 5. Conclusions

While weight reduction is associated with an improvement in glycemic control and lower cardiovascular risks in patients with T2DM, the increased mortality risk related to a lower BMI as well as the increased disability risk related to a higher BMI are also prominent, especially in older patients. The optimum BMI ranges and energy requirements for maintaining a healthy and sustainable BMI should be identified. In patients with T2DM, alterations in energy expenditure, such as a raised BMR, lowered TEF, and reduced levels of physical activity, have been reported. Limited evidence has shown that the TEE measured using the DLW method in patients with T2DM was not different from that of patients without diabetes, suggesting that the energy requirement data of subjects without diabetes may be applicable to these populations. LPA is one of the possible determinants of variability in the individual energy requirements of patients with T2DM, and the effect of increasing LPA on the improvement in nutritional intake and frailty prevention should be investigated further.

## Figures and Tables

**Table 1:** Total energy expenditure of patients with type 2 diabetes mellitus assessed using the doubly labeled water method.

Study (Ref)	Subjects	Age (Years)	BMI	TEE (kcal/Day)	TEE (kcal/kg-Weight/Day)	PAL
Chong et al., 1993 [43], 1995 [44] *	Patients with T2DM, 9 males/16 females	50.3 ± 11.4	29.8 ± 7.2	2877 ± 688	35.8 ± 6.4	1.78 ± 0.28
Salle et al., 2006 [15]	Patients with T2DM, *n* = 12		37.1 ± 4.67	3863 ± 1890		1.857 ± 0.503
	Patients without diabetes, *n* = 9		37.0 ± 3.40	3389 ± 887		1.881 ± 0.198
Yoshimura et al., 2019 [16]	Subjects with T2DM, 12 males	55 ± 7	24.0 ± 1.8	2490 ± 379	36.5 ± 5.0	
	Control subjects without diabetes, 10 males	55 ± 7	23.6 ± 1.8	2284 ± 243	33.7 ± 3.7	
Morino et al., 2019 [45]	Patients with T2DM, 28 males/24 females	70.2 ± 5.1	23.3 ± 3.0	2159 ^†^	36.4 ^†^	1.71 ^†^
	Patients without diabetes, 6 males/9 females	67.1 ± 4.7	22.7 ± 2.1	2168 ^†^	37.8 ^†^	1.81 ^†^
Ishikawa-Takata et al., 2020 [46]	Patients with T2DM, 5 males/4 females	51.7 ^†^	30.3 ^†^	2742 ^†^	34.4 ^†^	1.63 ^†^
	Subjects with IGT/IFG, 6 males/5 females	53.4 ^†^	30.6 ^†^	2901 ^†^	33.3 ^†^	2.00 ^†^
	Subjects with NGT, 5 males/5 females	53.8 ^†^	29.4 ^†^	2672 ^†^	32.6 ^†^	1.83 ^†^

BMI: body mass index, TEE: total energy expenditure, PAL: physical activity level; calculated as total energy expenditure/basal metabolic rate, T2DM: type 2 diabetes mellitus, IGT/IFG: impaired glucose tolerance and/or impaired fasting glucose, NGT: normal glucose tolerance. * Data were combined after omitting duplicates of the subjects. Data are expressed as mean ± SD or ^†^: median.

**Table 2:** The total energy expenditure, activity time, and estimated protein intake among subjects with type 2 diabetes mellitus with a low, middle, and high physical activity level (PAL).

	PAL			*p* Value ANCOVA
	Low Tertile	Mid Tertile	High Tertile	
Age (years)	70.5 ± 4.6	69.5 ± 5.7	70.5 ± 5.5	NS
Gender (male/female)	9/8	8/9	11/6	
BMI (kg/m^2^)	22.9 ± 3.1	23.3 ± 3.1	23.5 ± 2.8	NS
HbA1c (%)	6.8 ± 0.9	6.8 ± 0.5	7.0 ± 0.9	NS
PAL	1.54 ± 0.07	1.70 ± 0.04	1.90 ± 0.13	<0.001
TEE (kcal/day)	1905 ± 173	2164 ± 382	2426 ± 310	<0.001
TEE (kcal/kg-weight/day)	32.5 ± 2.8	36.4 ± 3.9	40.5 ± 5.9	<0.001
Accelerometry				
Sedentary time (min)	564 ± 85	496 ± 84	379±83	<0.001
Non-locomotive time				
LPA (min)	260 ± 66	312 ± 65	376 ± 64	<0.001
MVPA (min)	14 ± 32	29 ± 32	56 ± 32	0.0001
Locomotive time				
LPA (min)	28 ± 18	31 ± 18	44 ± 118	0.024
MVPA (min)	26 ± 37	23 ± 37	36 ± 36	0.594
Protein energy (%)	14.8 ± 1.9	15.5 ± 2.6	15.3 ± 3.6	NS
Protein intake (g/kg/day)	1.20 ± 0.19	1.41 ± 0.27	1.53 ± 0.45	0.013

Values are expressed as covariate-adjusted mean ± SD, adjusted for age, sex, and wearing time (in the case of accelerometry). *p* values were calculated after adjusting for age, sex, and wearing time (in the case of accelerometry). PAL: physical activity level, BMI: body mass index, TEE: total energy expenditure, LPA: low-intensity physical activity, MVPA: moderate-to-vigorous physical activity, NS: not significant.

## Data Availability

Not applicable.
